# Asymptomatic and submicroscopic malaria infections in sugar cane and rice development areas of Ethiopia

**DOI:** 10.21203/rs.3.rs-2692688/v1

**Published:** 2023-03-21

**Authors:** Hallelujah Getachew, Assalif Demissew, Ashenafi Abossie, Kassahun Habtamu, Xiaoming Wang, Daibin Zhong, Guofa Zhou, Ming-Chieh Lee, Elizabeth Hemming-Schroeder, Lauren Bradley, Teshome Degefa, Dawit Hawaria, Arega Tsegaye, James W. Kazura, Cristian Koepfli, Guiyun Yan, Delenasaw Yewhalaw

**Affiliations:** Department of Medical Laboratory Technology, Arbaminch College of Health Sciences, Arbaminch; Department of Medical Laboratory Sciences, College of Medicine and Health Sciences, Ambo University, Ambo; Department of Medical Laboratory Sciences, College of Medicine and Health Science, Arbaminch University, Arbaminch; Menelik II Medical & Health Science College, Addis Ababa; Program in Public Health, University of California at Irvine, Irvine, CA 92697; Program in Public Health, University of California at Irvine, Irvine, CA 92697; Program in Public Health, University of California at Irvine, Irvine, CA 92697; Program in Public Health, University of California at Irvine, Irvine, CA 92697; Center for Vector Born Infectious Diseases (CVID), Department of Microbiology Immunology and Pathology, Colorado State University; Program in Public Health, University of California at Irvine, Irvine, CA 92697; Department of Medical Laboratory Sciences, Institute of Health, Jimma University, Jimma; Hawassa University, School of Environmental Health, Hawassa; Department of Biology, College of Natural Science, Jimma University; Biomedical Research Case Western Reserve University, Cleveland, Ohio; Department of Biological Sciences 319 Galvin Life Sciences, Eck Institute for Global Health, University of Notre Dame, Notre Dame; Program in Public Health, University of California at Irvine, Irvine, CA 92697; Department of Medical Laboratory Sciences, Institute of Health, Jimma University, Jimma

**Keywords:** Irrigation, Submicroscopic malaria, P. ovale, Ethiopia

## Abstract

**Background:**

Water resource development projects such as dams and irrigation schemes have a positive impact on food security and poverty reduction but might result in increased prevalence of malaria.

**Methods:**

Two cross-sectional surveys were conducted in the dry and wet seasons in irrigated and non-irrigated clusters of Arjo sugarcane and Gambella rice development areas of Ethiopia in 2019. A total of 4464 and 2176 blood samples were collected from Arjo and Gambella. A subset of 2244 microscopy negative blood samples were analyzed by PCR.

**Results:**

Prevalence by microscopy was 2.0% (88/4464) in Arjo and 6.1% (133/2176) in Gambella. In Gambella, prevalence was significantly higher in irrigated clusters (10.4% vs 3.6%) than in non-irrigated clusters (*p* < 0.001), but no difference was found in Arjo (2.0% vs 2.0%; p = 0.993). Level of education was an individual risk factors associated with infection in Arjo [AOR: 3.2; 95%CI (1.27–8.16)] and in Gambella [AOR: 1.7; 95%CI (1.06–2.82)]. While duration of stay in the area for < 6 months [AOR: 4.7; 95%CI (1.84–12.15)] and being a migrant worker [AOR: 4.7; 95%CI (3.01–7.17)] were risk factors in Gambella. Season [AOR: 15.9; 95%CI (6.01–42.04)], no ITN utilization [AOR: 22.3; 95%CI (7.74–64.34)] were risk factors in Arjo, and irrigation [AOR: 2.4; 95%CI (1.45–4.07)] and family size [AOR: 2.3; 95%CI (1.30–4.09)] risk factors in Gambella. Of the 1713 and 531 randomly selected smear negative samples from Arjo and Gambella and analyzed by PCR the presence of Plasmodium infection was 1.2% and 12.8%, respectively. *P. falciparum, P. vivax,* and *P. ovale* were identified by PCR in both sites.

**Conclusion:**

Strengthening malaria surveillance and control in project development areas and proper health education for at-risk groups residing or working in such development corridors is needed.

## Introduction

Water resource development projects such as dams and irrigation schemes have a positive impact on food security and poverty reduction (1). However, such projects also alter ecosystems and increase mosquito abundance. They could result in a change in the malaria transmission pattern from seasonal to perennial in particular in areas with unstable malaria transmission (2, 3). The work opportunity in irrigation projects attracts a large number of migrant workers who might be at increased the risk of malaria. This is further exacerbated if migrant workers carry new strains such drug resistance parasites gene and also non-immune migrant works move to the area this may lead to malaria outbreak (4, 5).

Different agro-ecosystems and crop production types have an impact on mosquito proliferation, and consequently, the intensity of malaria transmission. Depending on the number of crop cycles, irrigation-based farming may also extend the mosquito breeding season and lengthen the malaria transmission period (6). Major crop irrigation schemes in Africa include rice, sugarcane, cotton, wheat and vegetables. Among them, rice grown in flooded irrigation provides potential larval habitats (7). Although a sugarcane plantation needs irrigation for maximum growth, it does not need to be flooded condition (8). Furthermore, *An. gambiae,* the major malaria vector in Africa, prefers environments with direct sunlight for breeding, thus the dense vegetation cover by sugarcane development would make the environment undesirable (6). However, poorly maintained water canals used for sugarcane irrigation may produce hatching grounds for mosquitoes (9). Studies have shown inconsistent results of malaria prevalence following the implementation of irrigation schemes. Several studies found that when compared to non-irrigated villages, the prevalence of malaria was increased in irrigated or dam areas (10–14). Other studies have shown decreased malaria prevalence in irrigated areas (6, 15, 16). Such discrepancies suggest that the effects of irrigation or dams on malaria transmission are poorly understood.

In malaria-endemic settings asymptomatic infections are abundant (17, 18). The occurrence of asymptomatic infections poses serious implications for malaria control and elimination programs (19, 20), as these individuals might carry gametocytes that contribute to the persistent transmission of malaria (21, 22). Often, the majority of asymptomatic infections are submicroscopic and can only be detected using molecular methods (23). In endemic areas, malaria elimination will not be feasible with the existing interventions alone (22, 24). Thus, additional strategies to detect asymptomatic carriers need to be considered. Therefore, the objective of the present study was to assess asymptomatic and submicroscopic malaria in sugarcane and rice development areas of Ethiopia

## Methods

### Study area

Data and blood samples were collected in sugarcane (Arjo) and rice (Gambella) development area of Ethiopia. The Arjo Didessa Sugarcane development irrigation is located in Jimma Arjo, Dabo Hanna and Bedele districts of East Wellega and Buno Bedele Zones of Oromia regional state. It is 540 km Southwest of Addis Ababa and is located at latitude 8.6° N, longitude 36.4°E, and; an altitude with a ranges of 1300 to 2280 m. The mean annual rainfall is 1477 millimeters, with bimodal rainfall (long rainy season June to September and short rainy season February to March) and with a relatively short dry season between December to January. The Saudi Star Agricultural Development PLC is located in Abobo district of Gambella region in West Ethiopia. It is 811km west of Addis Ababa located at latitude 7.9° N and longitude 34.5°E, and; an altitude with a ranges of 400–500m. The annual rainfall in Abobo is ranges between 900 to 1200 mm, with unimodal rainfall (long rainy season May to October) and long dry and hot season occurs between November-April (25).

**Figure 1.**
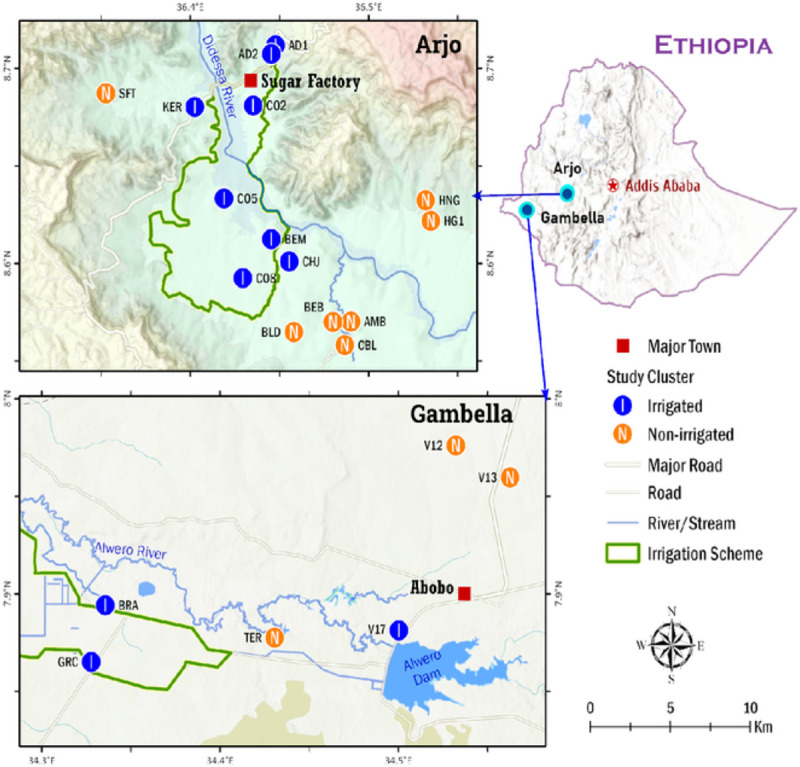
Map of the study sites

The total population of Jimma Arjo, Dabo Hanna and Bedele districts was 271,378. Thirteen *Kebeles* (the smallest administrative unit) were randomly selected from twenty six *Kebeles* surrounding Arjo Didessa Sugar cane irrigation scheme in the three districts with a catchment population of 51,407. These *Kebeles* were Abote-Didessa (AD), Ambelta (AMB), Bekelicha-Biftu (BEB), Beyima (BEM), Bildima-Daru (BLD), Chilalo-Bildima (CBL), Chefe-Jalala (CHJ), Hunde-Gudina (HNG), Kerka (KER), Sefra-Tabia (SFT), Command 2 (CO2), Command 5 (CO5), and Command 8 (CO8). From the above 13 *Kebeles*, fifteen (15) clusters were included for the mass blood surveys. A cluster was defined as a village within a *Kebele* comprising a minimum of 100 households and a maximum of 200 households. Two irrigated clusters were in Abote-Didessa (AD1, AD2), the others were KER, CHJ, BEM, CO2, CO5, and CO8. Non-irrigated cluster were AMB, BEB, BLD, CBL, HNG, HG1and SFT (Fig. 1). The sugarcane plantation has been irrigated by the use of sprinklers and in some part open gravity fed earthen ditches from Didessa River. In the non- irrigation clusters rain-fed farming took place, i.e. maize, sorghum, nut and peppers were grown at a subsistence level; and the villagers also keep cattle for their livelihood.

The total population of Abobo district was 26,080 living mainly on subsistence farming and fishing. From thirteen *Kebeles* that surround the Saudi Star rice irrigation, six *Kebeles* were randomly selected for this study. Irrigated clusters were in Saudi Star Bravo (BRA), Saudi Star GRC (GRC) and Village-17 (V17). Non-irrigated clusters were in Terkudi (TER), Village-12 (V12) and Village-13 (V13) (Fig. 1). The rice irrigation is supplied by open gravity-fed earthen ditches from the main canal of Alwero dam. The non-irrigation clusters used rain-fed farming, cotton, maize, sorghum and fishing.

### Demographic and parasitological surveys

Demographic data and blood samples were collected simultaneously in eight irrigated and seven non-irrigated clusters of Arjo and in three irrigated and three non-irrigated clusters of Gambella rice irrigation (Fig. 1.). Irrigated clusters were within 1 kilometer (km) radius and non-irrigated clusters were above 1 km radius from the edge of irrigated areas, considering *Anopheles* mosquito flight range (26). The spatial coordinates and demographic data were collected using Open Data Kit (ODK). Surveys were conducted in March 2019 (dry season) and October 2019 (wet season), representing low and high malaria transmission season, respectively. Households were randomly selected from each cluster to maximize coverage so that the surveyed population would represent each clusters. All consenting individuals living in the selected households were included in the study. Socio-demographic characteristics such as age, sex, occupation, place of residence, level of education, data on preventive measures of indoor residual spraying (IRS), insecticide treated net (ITN) ownership, type and village GPS coordinate were collected in both surveys. As well, malaria symptoms such as fever and any malaria related symptoms (chills, sweating, headache, vomit, and abdominal pain) during or 48 hours prior to blood collection. Study participant with body temperature above 35°c during or 48 hours prior to blood collection considered as febrile.

Capillary blood samples (300μL) were collected from all consenting participants for diagnosis by Rapid Diagnosis Tests (RDT), microscopy, and qPCR. For qPCR, dried blood spot (DBS) were prepared. The dried blood smear slides and the four dots of DBS samples were packed in slide box and plastic bag with desiccant, respectively. All samples were stored at room temperature then transported to Jimma University diagnostic and parasite culture laboratory for microscopic examination, DNA extraction and qPCR parasite determination.

### Sampling and sample size

The sample size (n) calculation was done using the formula for estimating single proportion at 95% confidence interval (CI), (Z α/2 = 1.96). To attained maximum sample size 50% was assumed for prevalence (P). A total of 384 household (n) were generated using 5% marginal error (d). Then by considering 4.9 household size in Oromia and 3.8 in Gambella region (27) the final number of study participants was calculated for each study site. Study households for cross-sectional parasitological survey (CPS) were identified through systematic sampling of every fifth house to obtain an average of 26 to 30 households for Arjo and 60 to 64 households for Gambella from each clusters depending on the total population size of the clusters.

### Blood film

Thick and thin blood films were prepared and stained with 10% Giemsa solution and examined under light microscope at Jimma University. Blood films were considered negative if no parasites were detected in 200 fields of the thick blood film. Thick blood films were used for detection and quantification of the parasites. Thin blood films were used for species identification.

Gametocytes and asexual stage parasites were counted against 200 and 500 white blood cells (WBCs), respectively, and densities (parasite/μl) was estimated using a factor of 8000 leukocytes/μl (28, 29). All positive slides and 10% of the negative slides were rechecked by a third technician.

### DNA extraction

Chelex-100 resin was used for DNA extraction following an established protocol (30). One dot of the DBS was cut into piece of approximately 3–5mm using a puncher. The puncher was cleaned by punching paper ten times after every sample. The blotted filter paper was transferred to 1.5ml Eppendorf tube then 950μl phosphate buffered saline (PBS) and 50 μl 10% Saponin were added for the lysis of RBCs. After mixing the sample, it was incubated at 4°c for 4 hours or overnight. The mixture was centrifuged at 14,000rpm for 10 minutes at room temperature and the supernatant was discarded. Any remnant of Saponin was removed by adding 1ml of PBS and centrifuge at 14,000rpm for about 5 minutes. The remaining PBS was discarded by centrifugation of the tube for about 15 seconds. The filter paper was left for 15 minutes to dry at room temperature. Subsequently, 150μl 20%Chelex suspension and 100μl distilled water was added to extract the parasite DNA by incubating the mixture at 95°c in a water bath for 10 minute with vortexing the mixture every 2 minutes during the process of incubation. Finally, the mixture was centrifuged at 14,000rpm for 1 minute and the 200μl supernatant (DNA) transferred to 0.5ml tube and stored at −20°C for PCR analysis.

### Plasmodium **species identification by qPCR**

Genomic DNA of each sample was amplified using 18S rRNA genes based primers. Amplification was performed in a 12μl PCR reaction mixture containing 2 μl of genomic DNA, 6μl PerrfeCTa (2X), 0.4 μl forward and reverse primers of *P. falciparum, P. vivax* and *P. ovale* each (F-F, F-R, Pv-1, Pv-2, Po-1, and Po-2) then 0.5μl Pf-fam, 0.5μl Pv-vic, and 0.5 μl Po-Ned for *P. falciparum, P. vivax* and *P. ovale* TaqMan probe in 0.1 double distilled water (31, 32) since there was *P. ovale* report in Northwest and Southeastern Ethiopia. Amplification reactions was performed in a 96 well Quant Studio 3, Applied Biosystems Real-Time PCR, with an initial denaturation at 50°C for 2 min and 95°C for 2 min, and 95°C for 3 sec, followed by 45 cycles at 60°Cfor 30 sec.

### Statistical analysis

Data was analyzed in JMP Pro (version 16 SAS Institute Inc., Cary, NC, USA). Univariate analysis was done on the associations between malaria prevalence and independent variables by regressing a single independent variable against *Plasmodium* positivity. Multivariate logistic regression run following stepwise backward selection of independent variables and removal of non-significant variables with *p* > 0.05. All significant variables remain in the final model and the model selection was based on the Akaike’s information criterion (AIC). The R statistical software package version (4.2.2) was used to compute W _*Mann−Whitney*_ test, bar graph and box plot. Model assumption was tested for each models.

## Result

Demographic information was obtained from 6640 individuals including 4464 (67.2%) from Arjo and 2176 (32.8%) from Gambella and can be found in Table 1.

### Parasite prevalence in Arjo

Of 4464 blood samples collected in Arjo, the overall malaria prevalence by microscopy was 2.0% (88/4464). The proportion of *Plasmodium* species by microscopy was 76.1% (67/88) *P. falciparum* and 23.9% (21/88) *P. vivax*.

**Figure 2.**
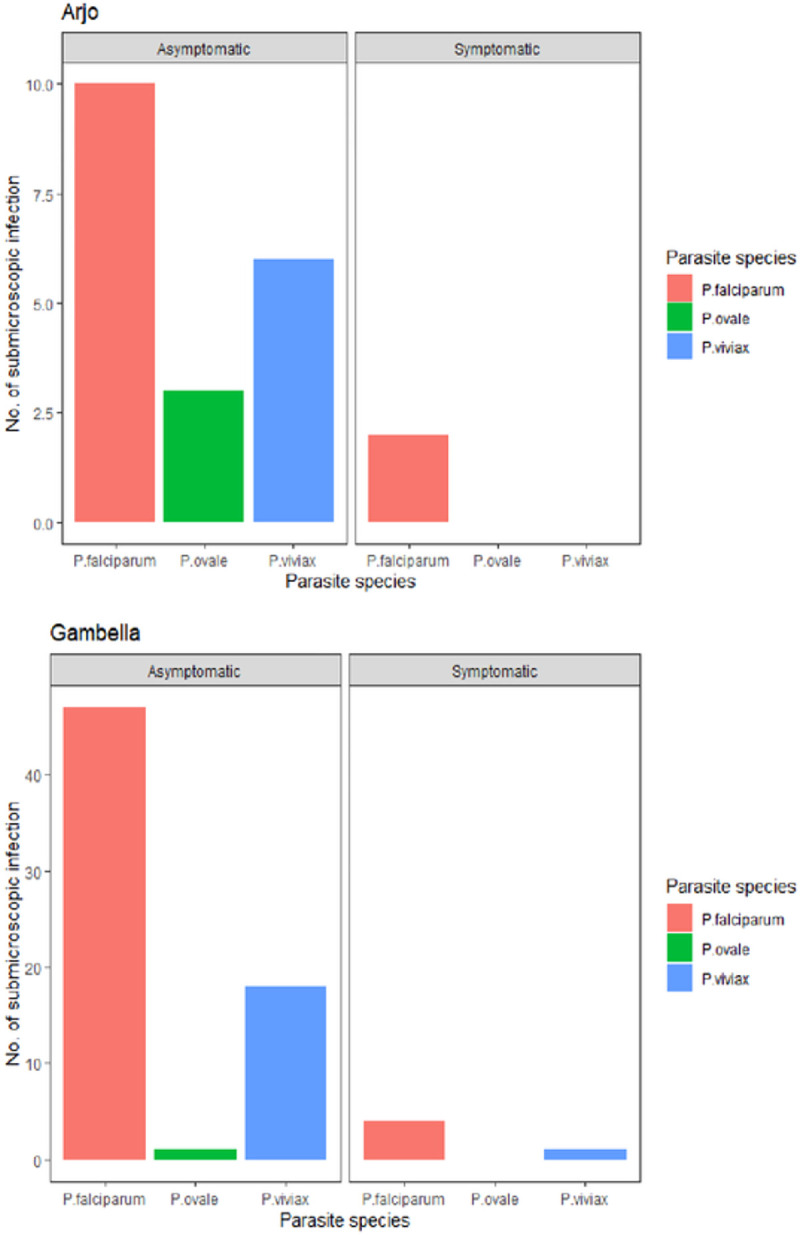
Proportion of submicroscopic infections among asymptomatic and symptomatic by parasite species in Arjo and Gambella, Ethiopia. (Individuals with malaria related symptoms or asymptotic during or 48h prior to mass blood survey).

One thousand seven hundred thirteen (1713) smear-negative samples were randomly selected for PCR analysis. Of the total samples analyzed by PCR, 1.2% (21/1713) carried submicroscopic infections. 90.5% (19/21) were afebrile and 9.5% (2/21) were febrile during or 48 h prior to the survey. The number of *Plasmodium* species in submicroscopic malaria infection was 12 *P. falciparum,* 6 *P. vivax,* and 3 *P. ovale* (Fig. 2).

Malaria prevalence by microscopy showed no significant difference between sugarcane irrigated 2.0% (44/2230) and in non-irrigated clusters 2.0% (44/2234) (p = 0.993).

The prevalence of malaria differs by season, in the wet season higher malaria prevalence was observed than in the dry season in both the sugarcane irrigated (wet: 3.3% (42/1253); dry: 0.2% (2/977); *p* = 0.0001) and non-irrigated clusters (wet: 2.9% (40/1372); dry: 0.5% (4/862); *p* = 0.0001). The prevalence of infection in the dry season in both sugarcane-irrigated and non-irrigated clusters was very low, 0.2% (2/977) and 0.5% (4/862) respectively (Table 2.).

**Figure 3.**
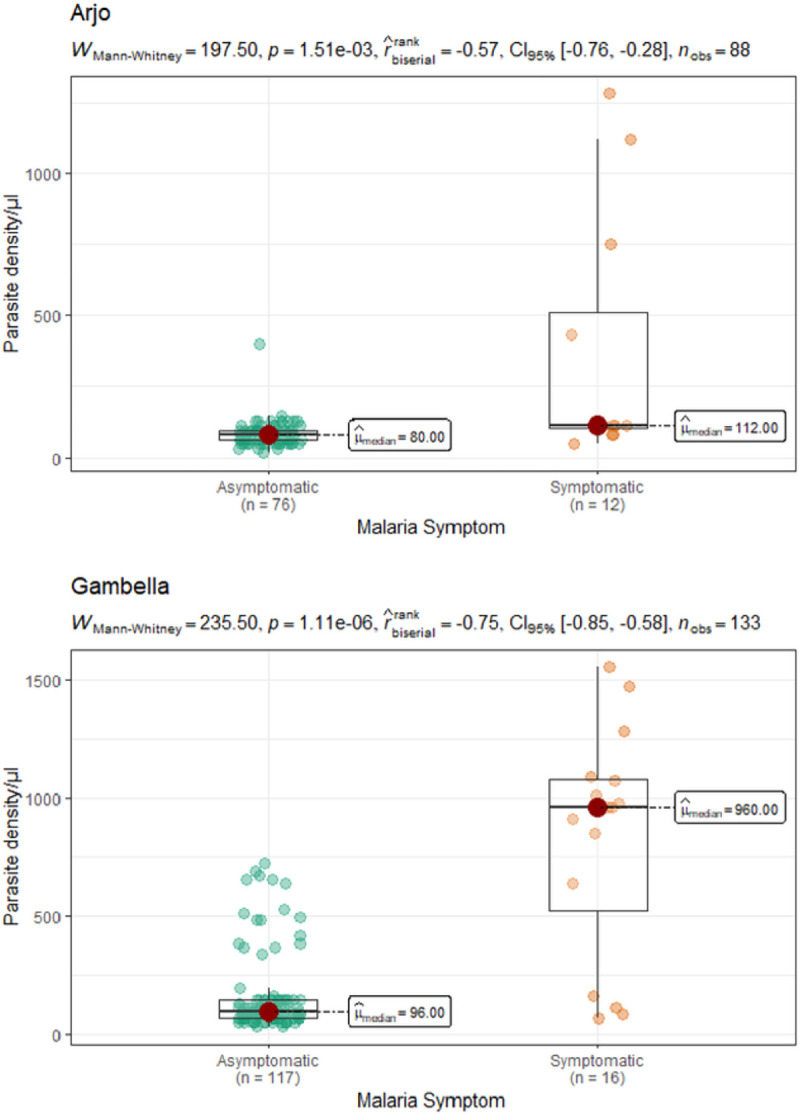
Parasite density in symptomatic and asymptomatic *Plasmodium*-infections by microscopy in Arjo and Gambella, Ethiopia.

There was significant difference in parasitemia load between symptomatic and asymptomatic *Plasmodium* infection by microscopy. The median parasite density/μl for symptomatic and asymptomatic infection in Arjo was: 112, IQR: 88–672 and 80, IQR: 64–96, respectively with *W _Mann–Whitney_* = 197.5, n1 = 12, n2 = 76, p = 1.51e-03) (Fig. 3.).

### Parasite prevalence in Gambella

The overall malaria prevalence by microscopy was 6.1% (133/2176) Gambella. The proportion of *Plasmodium* species by microscopy was 75.9% (101/133) *P. falciparum* and 24.1% (32/133) *P. vivax*.

Of the 531 smear-negative sub-samples collected from Gambella and analyzed by PCR, 12.8% (68/531) were submicroscopic malaria infection. 92.6% (63/68) were afebrile and 7.4% (5/68) were febrile. Among submicroscopic infections, 48 were *P. falciparum,* 16 *P. vivax,* 3 mixed (*P. falciparum* and *P. vivax*), and 1 *P. ovale* (Fig. 2.).

The prevalence of malaria was 10.4% (83/800) and 3.6% (50/1376) in rice irrigated and non-irrigated clusters, respectively (*p* = 0.0001, Table 2.). In rice irrigated clusters prevalence was higher during the dry season than in the wet season (12.9% (61/472) vs 6.7% (22/328); *p* = 0.0046). In non-irrigated clusters, marginally significant difference in malaria prevalence was observed between the wet season (4.9% (25/511)) and dry season (2.9% (25/865), *p* = 0.055, Table 2).

There was significant difference in parasitemia load between symptomatic and asymptomatic *Plasmodium* infection by microscopy. The median parasite density/μl for symptomatic and asymptomatic infection in Gambella was: 960, IQR: 280–1084 and 96, IQR: 64–144, respectively with W _*Mann–Whitney*_ = 235.5, n1 = 16, n2 = 117, *p* = 1.11e-06) (Fig. 3.).

### **Univariate and multivariate analysis of risk factors for** Plasmodium **infection**

In Arjo, sex, age group, duration of stay in the area, and migrant worker status were not significantly associated with infection. Individuals who were not office workers had significantly higher malaria infection rates than office workers [OR: 5.24, 95%CI: (1.47–18.68), *p* = 0.010]. Also individuals who never attended school had higher malaria infection than those with higher education [OR: 3.22, 95%CI: (1.27–8.18), *p* = 0.014] (Table 3.). Stepwise backward elimination of independent variables with the highest *p*-value resulted in the final model, in which only level of education remained significant predictors of *Plasmodium* infection with full model AIC = 871.8 and in the final model AIC = 863.5 (Table 4.).

In Arjo, household risk factors such as irrigation status, family size, roof material, floor material, wall materials, number of sleeping rooms in the house, number of LLINs in the house, and IRS were not associated with infection. In contrast, season and LLINs utilization were risk factors for malaria infection. Wet season [OR: 5.99, 95%CI (2.33, 15.41), *p* = 0.0001] and not utilizing LLIN [OR: 8.55, 95%CI (3.00, 24.34), *p* = 0.0001] were significantly associated with infection (Table 5.). Stepwise backward elimination of independent variables with the highest *p*-value resulted in the final model, in which season and LLIN utilization significant predictors of infection with full model AIC = 317 and in the final model AIC = 300 (Table 6.).

In Gambella, being a rice plantation worker [OR: 3.88, 95%CI: (1.60–9.40), *p* = 0.0026], age group < 15 [OR: 0.41, 95%CI: (0.20–0.83), *p* = 0.013], primary educational status [OR: 0.52, 95%CI: (0.33–0.82), *p* = 0.0046], being a preschool child [OR = 0.21, 95%CI: (0.09–0.51), *p* = 0.0005], < 6 months [OR: 3.29, 95%CI: (1.51–7.21), *p* = 0.0028], being a migrant worker [OR: 5.18, 95%CI: (3.53–7.59), *p* = 0.0001] were associated with *Plasmodium* infection (Table 3.). Sex was not significantly associated with *Plasmodium* infection. In the final model, level of education, duration of stay in the area and migrant worker significant predictors of *Plasmodium* infection with full model AIC = 925.3 and in the final model AIC = 920.5 (Table 4.)

In Gambella, household risk factors such as family size, roof material, number of sleeping rooms in the house, number of LLIN per household and a house that was treated with IRS in the preceding 12 months and season were not associated with *Plasmodium* infection in the univariate analysis. Whereas, irrigation [OR: 2.42, 95%CI, (1.49–3.91), *p* = 0.0003], mud/earth floor material [OR: 0.55, 95%CI, (0.35–0.88), *p* = 0.0118], corrugated iron wall material [OR: 1.77, 95%CI (1.12–2.79), *p* = 0.0142] and no LLIN utilization [OR: 2.10, 95%CI, (1.27–3.18), *p* = 0.0029] were significantly associated with infection (Table 5.). Stepwise backward elimination of independent variables with the highest *p*-value resulted in the final model with full model AIC = 514.7 and in the final model AIC = 505.1. Households located in irrigated clusters and HH with a family size of > 5 were significant predictors of *Plasmodium* infection. Even if, number of LLIN per HH in the final model it was not significantly associated to HH *Plasmodium* infection (Table 6.).

## Discussion

In Gambella, prevalence by microscopy was higher in irrigated vs. non-irrigated clusters, while no difference was observed in Arjo. In Arjo, prevalence was higher in the wet season, while this was the case only for non-irrigated sites in Gambella, and the effect was reversed in irrigated sites. Among the individual risk factors, educational level was significantly associated with *Plasmodium* infection in Arjo and Gambella. Whereas, duration of stay in the area, and being a migrant worker were significantly associated with *Plasmodium* infection in Gambella. Known household factors such LLINs utilization status, and high transmission season were significantly associated with *Plasmodium* infection in Arjo. In Gambella, irrigation and family size of the households were significantly associated with *Plasmodium* infection.

Asymptomatic sub-microscopic malaria is a major obstacle to malaria elimination due to its being a hidden reservoir and facilitate onward transmission. Therefore, malaria elimination requires targeting sub-microscopic carriers (23). Several studies showed a range of PCR prevalence in low transmission areas from 0 to 16.8 and 16.3 to 82.5 in high transmission areas respectively (18, 33–36). In our study, the prevalence of sub-microscopic *Plasmodium* infection was 1.2% in Arjo (low transmission setting) whereas, 12.8% in Gambella (high transmission setting). Studies in Ethiopia showed different prevalence of sub-microscopic malaria 2.4% (37), 3.3% (38), 9.7% (19),12.7% (39), 18.7% (40) and 19.2% (41). In our study (Arjo site), the low prevalence of sub-microscopic malaria infection might be due to the intensive use of primaquine (PQ) since 2018 targeting the elimination of malaria in low transmission settings.

*Plasmodium* ovale was reported in both study sites for the first time. All infections were submicroscopic. Correct identification of parasite species and parasite distribution are important to facilitate proper diagnosis, treatment, case management and malaria elimination. Previously *P. ovale* was reported in Ethiopia in 1969, but it was not reported until 2013 and 2015 from northwestern (42) and southeastern Ethiopia (43). Two recent serological studies showed that there is evidence of the presence of *P. ovale* and *P. malariae* in different parts of the country (38, 44).

In Arjo, significant increment of malaria prevalence was not observed between sugarcane-irrigated and non-irrigated clusters however, rice irrigation showed 3-fold increase in malaria infection than non-irrigated clusters. Moreover, malaria infection doubled in the low transmission season (12.9%) than high transmission season (6.7%) in rice irrigation clusters. Similar findings were also documented in two studies from east-central Tanzania (12, 14). On the other hand, high malaria prevalence was recorded in sugarcane irrigated than rice irrigated in northern Tanzania (15). The discrepancy of the results might be due to the majority of study population were migrant workers in Tanzanian study.

Also, studies conducted in our study sites demonstrated that anopheline mosquito abundance was higher in rice-irrigated clusters than non-irrigated clusters and the sporozoite rate in rice-irrigated clusters was 10-fold higher than non-irrigated clusters in Gambella (45). Whereas, in Arjo, the anopheline mosquito density was 7 fold higher in sugarcane-irrigated clusters than in non-irrigated clusters during wet season (46). This could be the odor emanates from rice strongly attracts the female anopheline mosquitoes oviposition than the sugar cane (47, 48) and the microhabitats formed by the flooded type of rice irrigation is favorable for mosquito proliferation during the low malaria transmission season. However, in Arjo sugarcane plantation, large proportion of irrigation system was sprinkler water for seedlings. Thus, the result might suggest that rice irrigation had higher contribution for persistent malaria transmission in the area.

In the present study, prevalence of infection was 16-fold higher in the wet season compared to dry season. This finding was complemented by an entomological study that showed higher mosquito abundance in wet season than the dry season in Arjo (46). In addition, a retrospective study which was conducted in Arjo showed higher malaria cases in wet season (49). Variation in the prevalence of malaria in terms of season in irrigated sites was observed in other studies (11, 14, 25, 50, 51). The difference might be due to the type of irrigation and type of crop cultivated.

Vector control is one of the most cost effective tool in reducing malaria transmission. This includes spraying IRS and ITN utilization. Previously high coverage of IRS was done by the African IRS programme funded by USAID-PMI until 2010. At the moment, IRS is conducted by the national malarial elimination program of the Ethiopian government and the coverage was limited and targeted to malaria hotspot areas. However, IRS coverage was higher 37.6% in Arjo area compared to the 2015 regional coverage 29.8% (54). Similarly, IRS coverage was double (42.4% ) in Gambella than 2015 regional coverage 21.5% (54). The mass blood survey was conducted seven months after IRS sprayed and the protective effect might be reduced due to dosage concentration, duration, and other factors could affect the protective efficacy of IRS. The protective effect of the IRS was not as effective as expected in this study.

Although, LLINs utilization was 32.2% in 2017 (53) and 40.3% in 2020 (54), poor utilization and no utilization of LLINs still significantly associated with *Plasmodium* infection in both irrigated and non-irrigated clusters in Arjo. The risk of *Plasmodium* infection was 10-fold increase in households who had a habit of poorly utilization of LLIN than households who used persistently. Additionally, households that did not utilize LLIN were 22 times at higher risk of *Plasmodium* infection than persistently utilizing in Arjo. This result is in line with the results of different studies (14, 55–57). In both Arjo and Gambella studies showed that those who never attended school were 3.2 and 1.7 times at higher risk of *Plasmodium* infection than those who had secondary and above education level respectively. Our finding is in consistent with findings from other similar studies (58–60). Level of education had direct associated on the knowledge and utilization of malaria prevention tools. While preschool age children were 81% less likely to acquire *Plasmodium* infection than higher educational level [AOR: 0.19, 95%CI: (0.07–0.53) *p* = 0.0016] in Gambella study. Our findings indicates that poor LLIN utilization could be correlated with the level of education.

Newly migrant population is more prone to malaria infection due to different reasons. In our study, being a migrant worker were 4.7 times more prone to malaria infection than the non- migrant residents. In addition, migrant workers who stayed in the area for < 6 months and 7 to 12 months were 4.7 and 5.6 times at risk of malaria infection than those who stayed for more than three years, respectively. Our finding is in line with findings from several studies on migrant workers involved in commercial agricultural activities (5, 55, 58–61). In the era of malaria elimination human mobility has to be in account as a malaria risk group due to these groups might carry new strain, drug resistance parasite gene and if they travel from non-endemic area to endemic region this can cause an outbreak. One of the drawback of the 1950s and 1960s malaria eradication campaigns was unable to consider human mobility (62). Plasmodium infection in Gambella was also significantly associated with family size. Similar finding were observed in country wide study in Ethiopia (63) and India (64, 65). There is a possibility when the number of resident in a household increases, the olfactory cues to attract the anopheline mosquito become stronger and increases the chance to be bitten by the vector (66).

## Conclusion

The co-existence of *P. vivax, P. ovale* and *P. falciparum* coupled with the high malaria prevalence recorded in irrigated areas indicated the significant impact of irrigation schemes on malaria transmission in the study areas. Poor ITN utilization being illiterate and population movement were the main malaria risk factors in the area. In addition, staying in irrigated less than 12 months and having a family size more than 5 were malaria risk factors. Therefore, strengthening malaria control and elimination; including proper utilization of vector control tools and health education for migrant population, should be implemented in water resource development projects such as irrigation schemes.

## Figures and Tables

**Table 1:** Demographic characteristics of the study participants (March 2019 and October2019)

	Arjo			Gambella		
Characteristic	Irrigated (%)	Non-irrigated (%)	*P-value*	Irrigated (%)	Non-irrigated (%)	*P-value*
Sex						
Male	1071 (24.0)	999(22.4)	0.0266	485(22.3)	693 (31.8)	0.0001
Female	1159 (25.9)	1235 (27.7)		315(14.5)	683 (31.4)	
Age groups:						
< 5	259 (5.8)	376 (8.4)	0.0001	65(3.0)	178 (8.2)	0.0001
5_14	483 (10.8)	560 (12.5)		142(6.5)	395 (18.2)	
15_29	958 (21.5)	720 (16.1)		350(16.1)	361 (16.6)	
30_45	398 (8.9)	383 (8.6)		173(7.9)	307 (14.1)	
> 45	132 (3.0)	195 (4.4)		70(3.2)	135 (6.2)	
Education level						
≥ Secondary	447 (10.0)	141 (3.2)	0.0001	268(12.3)	209 (9.6)	0.0001
Primary	757 (17.0)	625 (14)		301 (13.8)	537 (24.7)	
Preschool age^€^	374 (8.4)	522 (20.1)		86(4.0)	219 (10.1)	
Never attended school	652 (14.6)	946 (21.2)		145(6.7)	411 (18.9)	
Occupation:						
Office worker	200 (4.5)	109 (2.4)	0.0001	63(2.9)	51 (2.3)	0.0001
Non-schooled child	410 (9.2)	622 (13.9)		92 (4.2)	244 (11.2)	
Student	438(9.8)	426 (9.5)		190 (8.7)	434 (20.0)	
Agriculture laborer	641 (14.4)	1032 (23.1)		163 (7.5)	586 (26.9)	
Factory workers^¥^	520(11.7)	3 (0.1)		235 (10.8)	13 (0.6)	
Others^¶^	21(0.5)	42 (0.9)		57 (2.6)	48 (2.2)	
Migrant worker						
No	2014 (45.1)	2193 (49.1)	0.0001	553 (25.4)	1374 (63.1)	0.0001
Yes	216 (4.8)	41 (0.9)		247 (11.4)	2 (0.1)	
Duration of stay in the area						
> 3 years	1662 (37.2)	1905 (42.7)	0.0001	718 (33.0)	1257 (57.8)	0.1418
1–3 years	278 (6.2)	175 (3.9)		42 (1.9)	45 (2.1)	
7–12 months	114 (2.6)	95 (2.1)		22 (1.0)	44 (2.0)	
< 6 months	176 (3.9)	59 (1.3)		18 (0.8)	30 (1.4)	
Season						
Dry	977 (21.9)	862 (19.3)	0.0004	472 (21.7)	865 (39.7)	0.0742
Wet	1253 (28.1)	1372 (30.7)		328 (15.1)	511 (23.5)	

¥: includes sugarcane and rice plantation workers

¶: drivers, miners and fisherman

€: children less than 7 years old not yet start formal education

**Table 2:** Malaria infection in irrigated and non-irrigated clusters in Arjo sugar development scheme and Gambella Rice irrigation, Ethiopia (October 2019 and March 2019)

		Arjo		Gambella	
Season		Total examined	Microscopy Positive	Total examined	Microscopy Positive
Irrigated	Low transmission	977	2 (0.2%)	472	61 (12.9%) **
	High transmission	1253	42 (3.3%) ***	328	22 (6.7%)
	Total	2230	44 (2.0%)	800	83 (10.4%)
Non-irrigated	Low transmission	862	4 (0.5%)	865	25 (2.9%)
	High transmission	1372	40 (2.9%) ***	511	25 (4.9%)
	Total	2234	44 (2.0%)	1376	50 (3.6%)

** *p* < = 0.01

*** *p* < 0.001

**Table 3:** Univariate analysis of individual risk factors and malaria infection by microscopy, Ethiopia (March 2019 and October 2019)

Individual risk factor		Arjo				Gambella			
		Prevalence	Case			Prevalence	Case		
		(%)	n	N	OR (95%CI)	(%)	n	N	OR (95%CI)
Sex	Female	1.05	47	2394	1	2.39	52	998	1
	Male	0.92	41	2070	1.01(0.66,1.54)	3.72	81	1178	1.34(0.94,1.92)
Age groups	< 15	0.74	33	1678	0.80(0.37,1.75)	0.97	21	780	0.41 (0.20,0.83)**
	15_45	1.05	47	2459	0.78(0.36,1.66)	4.55	99	1191	1.34(0.74,2.43)
	> 45	0.18	8	327	1	0.60	13	205	1
Occupation	Office worker	0.11	5	309	1	0.28	6	114	1
	Student	0.31	14	864	1.00(0.36,2.80)	0.78	17	624	0.50(0.19,1.31)
	Non-school child	0.45	20	1032	1.20(0.45,3.23)	0.32	7	336	0.38(0.12,1.16)
	Agricultural laborer	0.83	37	1673	1.38(0.54,3.53)	2.16	47	749	1.20(0.50,2.88)
	Factory worker^¥^	0.16	7	523	0.82(0.26,2.62)	2.02	44	248	3.88(1.60,9.40)***
	Others^¶^	0.11	5	63	5.24(1.47,18.68)**	0.55	12	105	2.32(0.84,6.43)
Education status	≥ Secondary	0.11	5	588	1	1.88	41	477	1
	Primary	0.49	22	1382	1.89(0.71,5.00)	1.79	39	838	0.52(0.33, 0.82)***
	Preschool age^€^	0.40	18	896	2.39(0.88,6.47)	0.28	6	305	0.21(0.09, 0.51)***
	Never attended school	0.96	43	1598	3.22(1.27, 8.18)0.01	2.16	47	556	0.98(0.63, 1.52)
Duration of stay in the area	> 3 years	1.50	67	3567	1	5.19	113	1975	1
	1–3 years	0.18	8	453	0.94(0.45,1.97)	0.28	6	87	1.22(0.52,2.86)
	7–12 months	0.18	8	209	2.08(0.98,4.39)	0.28	6	66	1.65(0.70,3.89)
	≤ 6 months	0.11	5	235	1.13(0.45,2.84)	0.37	8	48	3.29(1.51,7.21)***
Migrant worker	No	1.88	84	4207	1	3.91	85	1927	1
	Yes	0.09	4	257	0.78(0.28,2.13)	2.21	48	249	5.18(3.53,7.59)***

* *p* < 0.05

** *p* < = 0.01

*** *p* < 0.001

¥: includes sugarcane and rice plantation workers

¶: drivers, miners and fisherman

€: children less than 7 years old not yet start formal education

**Table 4:** Multivariate analysis of individual risk factor and malaria infection by microscopy in Arjo and Gambella, Ethiopia (March 2019 and October 2019)

Individual risk factor		Prevalence	Case		
		(%)	n	N	AOR (95%CI)
Arjo	**Education status**				
	≥ Secondary	0.11	5	588	1
	Primary	0.49	22	1382	1.89(0.71–5.00)
	Preschool age€	0.40	18	896	2.39(0.88–6.47)
	Never attended school	0.96	43	1598	3.22(1.27–8.18)**
Gambella	**Education status**				
	≥ Secondary	1.88	41	477	1
	Primary	1.79	39	838	0.74(0.46–1.19)
	Preschool age€	0.28	6	305	0.19(0.07–0.53)***
	Never attended school	2.16	47	556	1.73(1.06–2.83)**
	**Duration of stay in the area**				
	> 3 years	5.19	113	1975	1
	1–3 years	0.28	6	87	2.16(0.85–5.52)
	7–12 months	0.28	6	66	5.55(1.90–16.18)***
	< 6 months	0.37	8	48	4.72(1.83–12.14)***
	**Migrant worker**				
	No	3.91	85	1927	1
	Yes	2.21	48	249	4.66(3.02–7.18)***

* *p* < 0.05

** *p* < = 0.01

*** *p* < 0.001

€: children less than 7 years old not yet start formal education

**Table 5:** Univariate analysis of household risk factor by microscopy, Ethiopia (March 2019 and October 2019)

Household risk factor		Arjo				Gambella			
		Prevalence	Case			Prevalence	Case		
		(%)	n	N	OR (95%CI)	(%)	n	N	OR (95%CI)
Irrigation Status	Non-irrigated	1.16	21	822	1	3.95	27	335	1
	Irrigated	0.94	17	986	0.67(0.35,1.28)	8.92	61	349	2.42(1.49,3.91)***
Season	Dry	0.28	5	847	1	7.16	49	389	1
	Wet	1.83	33	961	5.99(2.33,15.41)***	5.70	39	295	1.06(0.67,1.66)
Family size	1_5	1.27	23	1226	1	9.21	63	528	1
	> 5	0.83	15	582	1.38(0.72,2.67)	3.65	25	156	1.41(0.85,2.33)
Roof material	Corrugated iron	1.44	26	1215	1	8.77	60	417	1
	Grass (thatch)	0.66	12	593	0.94(0.47,1.89)	4.09	28	267	0.70(0.43,1.12)
Floor material	Cement	0.11	2	94	1	5.41	37	208	1
	Mud/earth	1.99	36	1714	0.99(0.23,4.16)	7.46	51	476	0.55(0.35,0.88)**
Wall material	Mud	1.77	32	1446	1	7.31	50	467	1
	Corrugated iron	0.33	6	362	0.74(0.31,1.79)	5.56	38	217	1.77(1.12,2.79)**
Number of sleeping rooms	≥ Three	0.28	5	230	1	0.73	5	47	1
	Two	1.11	20	799	1.16(0.43,3.11)	2.19	15	139	1.02(0.35,2.96)
	One	0.72	13	779	0.76(0.27,2.17)	9.94	68	498	1.33(0.51,3.48)
Number of LLIN per household	≥Three	0.28	5	271	1	0.73	5	47	1
	Two	0.50	9	531	0.92(0.30,2.76)	1.17	8	156	0.45(0.14,1.46)
	One	0.39	7	523	0.72(0.23,2.29)	5.26	36	265	1.32(0.49,3.56)
	Not available	0.94	17	483	1.94(0.71,5.32)	5.70	39	216	1.85(0.69,4.98)
LLIN usage per HH	Always	0.22	4	773	1	6.29	43	438	1
	Sometime	0.17	3	307	1.90(0.42,8.53)	0.44	3	12	3.06(0.80,11.74)
	Never	1.71	31	728	8.55(3.00,24.34)***	6.14	42	234	2.01(1.27,3.18)**
IRS sprayed the past 12 months	Yes	0.61	11	680	1	5.85	40	291	1
	No	1.49	27	1128	1.49(0.73,3.03)	7.02	48	393	0.87(0.56,1.37)

* *p* < 0.05

** *p* < = 0.01

*** *p* < 0.001

**Table 6:** Multivariate logistic regression of household risk factors between irrigated and non-irrigated clusters by microscopy in Arjo and Gambella, Ethiopia (March 2019 and October 2019)

Household risk factor		Prevalence	Case		
		(%)	n	N	AOR (95%CI)
Arjo	**Season**				
	Dry	0.28	5	847	1
	Wet	1.83	33	961	15.90(6.01,42.04)***
	**LLIN Usage per household**				
	Always	0.22	4	773	1
	Sometime	0.17	3	307	10.15(2.15,47.88)***
	Never	1.71	31	728	22.32(7.74,64.34)***
Gambella	**Irrigation Status**				
	Non-irrigated	3.95	27	335	1
	Irrigated	8.92	61	349	2.43(1.45, 4.07)***
	**Family size**				
	1_5	9.21	63	528	1
	> 5	3.65	25	156	2.30(1.30, 4.09)***
	**Number of LLIN per household**				
	≥Three	0.73	5	47	1
	Two	1.17	8	156	0.49(0.15, 1.61)
	One	5.26	36	265	1.53(0.53, 4.45)
	Not available	5.70	39	216	1.88(0.65, 5.39)

* *p* < 0.05

** *p* < = 0.01

*** *p* < 0.001

## Data Availability

The datasets used and/or analyzed during the current study are available from the corresponding author on reasonable request.

## References

[1] Lipton M, Litch eld J, Faurès JM. The effects of irrigation on poverty: A framework for analysis. Water Policy. 2003;5(5–6):413–27.

[2] Keiser J, De Castro MC, Maltese MF, Bos R, Tanner M, Singer BH, Effect of irrigation and large dams on the burden of malaria on a global and regional scale. Am J Trop Med Hyg. 2005;72(4):392–406. 15827275

[3] Kibret S, Lautze J, McCartney M, Wilson GG, Nhamo L. Malaria impact of large dams in sub-Saharan Africa: maps, estimates and predictions. Malar J. 2015;14(1):1–12. 26337834 10.1186/s12936-015-0873-2 PMC4560078

[4] Oaks SC, Mitchell VS, Pearson GW and CC. Malaria: Obstacles and Opportunities (1991). NATIONAL ACADEMY PRESS Washington, D.C; 1991. 1–309 p. 25121285

[5] Ijumba JN, Shenton FC, Clarke SE, Mosha FW, Lindsay SW. Irrigated crop production is associated with less malaria than traditional agricultural practices in Tanzania. Trans R Soc Trop Med Hyg. 2002;96(5):476–80. 12474470 10.1016/s0035-9203(02)90408-6

[6] Ijumba JN, Lindsay SW. Impact of irrigation on malaria in Africa: Paddies paradox. Med Vet Entomol. 2001;15(1):1–11. 11297093 10.1046/j.1365-2915.2001.00279.x

[7] Service MW. Rice, a challenge to health. Parasitol Today. 1989;5(5):162–5. 15463204 10.1016/0169-4758(89)90083-5

[8] Arnon I. Crop production in dry regions. Exp Agric. 1973;9(1).

[9] Chimbari MJ, Chirebvu E, Ndlela B. Malaria and schistosomiasis risks associated with surface and sprinkler irrigation systems in Zimbabwe. Acta Trop. 2004;89:205–13. 14732242 10.1016/j.actatropica.2003.09.015

[10] Yewhalaw D, Legesse W, Van Bortel W, Gebre-Selassie S, Kloos H, Duchateau L, Malaria and water resource development: The case of Gilgel-Gibe hydroelectric dam in Ethiopia. Malar J. 2009;8(1):1–10. 19178727 10.1186/1475-2875-8-21 PMC2649153

[11] Kibret S, Alemu Y, Boelee E, Tekie H, Alemu D, Petros B. The impact of a small-scale irrigation scheme on malaria transmission in Ziway area, Central Ethiopia. Trop Med Int Heal. 2010;15(1):41–50. 10.1111/j.1365-3156.2009.02423.x 19917039

[12] Mboera LEG, Senkoro KP, Rumisha SF, Mayala BK, Shayo EH, Mlozi MRS. Plasmodium falciparum and helminth coinfections among schoolchildren in relation to agro-ecosystems in Mvomero District, Tanzania. Acta Trop. 2011;120(1–2):95–102. 21741929 10.1016/j.actatropica.2011.06.007

[13] Jaleta KT, Hill SR, Seyoum E, Balkew M, Gebre-Michael T, Ignell R, Agro-ecosystems impact malaria prevalence: Large-scale irrigation drives vector population in western Ethiopia. Malar J. 2013;12(1):1–11. 24083353 10.1186/1475-2875-12-350 PMC3850965

[14] Rumisha SF, Shayo EH, Mboera LEG. Spatio-temporal prevalence of malaria and anaemia in relation to agro-ecosystems in Mvomero district, Tanzania. Malar J. 2019;18(1):1–14. 31288840 10.1186/s12936-019-2859-y PMC6617584

[15] Ijumba JN, Mosha FW, Lindsay SW. Malaria transmission risk variations derived from different agricultural practices in an irrigated area of northern Tanzania. Med Vet Entomol. 2002;16(1):28–38. 11963979 10.1046/j.0269-283x.2002.00337.x

[16] Sissoko MS, Dicko A, Briët OJT , Sissoko M, Sagara I, Keita HD, Sogoba M, Rogier C, Touré YT DO. Malaria incidence in relation to rice cultivation in the irrigated Sahel of Mali. 2004;89:161–70. 10.1016/j.actatropica.2003.10.015 14732238

[17] Eke RA, Chigbu LN, Nwachukwu W. High prevalence of asymptomatic plasmodium infection in a suburb of Aba Town, Nigeria. Ann Afr Med. 2006;5(1):42–5.

[18] Lindblade KA, Steinhardt L, Samuels A, Kachur SP, Slutsker L. The silent threat: Asymptomatic parasitemia and malaria transmission. Expert Rev Anti Infect Ther. 2013;11(6):623–39. 23750733 10.1586/eri.13.45

[19] Tadesse FG, Pett H, Baidjoe A, Lanke K, Grignard L, Sutherland C, Submicroscopic carriage of Plasmodium falciparum and Plasmodium vivax in a low endemic area in Ethiopia where no parasitaemia was detected by microscopy or rapid diagnostic test. Malar J. 2015;14(1):1–7. 26242243 10.1186/s12936-015-0821-1 PMC4524028

[20] Koepfli C, Ome-Kaius M, Jally S, Malau E, Maripal S, Ginny J, Sustained malaria control over an 8-year period in Papua New Guinea: The challenge of low-density asymptomatic plasmodium infections. J Infect Dis. 2017;216(11):1434–43. 29029179 10.1093/infdis/jix507 PMC5853328

[21] Ouédraogo AL, Gonçalves BP, Gnémé A, Wenger EA, Guelbeogo MW, Ouédraogo A, Dynamics of the human infectious reservoir for malaria determined by mosquito feeding assays and ultrasensitive malaria diagnosis in Burkina Faso. J Infect Dis. 2016;213(1):90–9. 26142435 10.1093/infdis/jiv370

[22] Vallejo AF, García J, Amado-Garavito AB, Arévalo-Herrera M, Herrera S. Plasmodium vivax gametocyte infectivity in submicroscopic infections. Malar J. 2016;15(1):1–9. 26822406 10.1186/s12936-016-1104-1 PMC4730736

[23] Lin JT, Saunders DL, Meshnick SR. The role of submicroscopic parasitemia in malaria transmission: What is the evidence? Trends Parasitol. 2014;30(4):183–90. 24642035 10.1016/j.pt.2014.02.004 PMC4049069

[24] Coalson JE, Walldorf JA, Cohee LM, Ismail MD, Mathanga D, Cordy RJ, High prevalence of Plasmodium falciparum gametocyte infections in school-age children using molecular detection: patterns and predictors of risk from a cross-sectional study in southern Malawi. Malar J. 2016;15(1):1–17. 27809907 10.1186/s12936-016-1587-9 PMC5096312

[25] Haileselassie W, Parker DM, Taye B, David RE, Zemene E, Lee MC, Burden of malaria , impact of interventions and climate variability in Western Ethiopia : an area with large irrigation based farming. BMC Public Health. 2022;1–11. 35093055 10.1186/s12889-022-12571-9 PMC8800266

[26] Service MW, Place P. Mosquito ( Diptera : Culicidae ) Dispersal — The Long and Short of It. 2018;34:579–88. 10.1093/jmedent/34.6.579 9439109

[27] Federal Democratic Republic of Ethiopia Population Census Commission. Summary and Statistical Report of the 2007 Population and Housing Census. 2008.

[28] World Health Organization (WHO). Basic malaria microscope. Part 1 learner’s guide. 2nd ed. Geneva, Switzerland: World Health Organization; 2010. 1–80 p.

[29] Laman M, Moore BR, Benjamin J, Padapu N, Tarongka N, Siba P, Comparison of an assumed versus measured leucocyte count in parasite density calculations in Papua New Guinean children with uncomplicated malaria. Malar J. 2014;13(1):1–6. 24739250 10.1186/1475-2875-13-145 PMC3991873

[30] Wooden J, Kyes S, Sibley CH. PCR and strain identification in Plasmodium falciparum. Parasitol Today. 1993;9(8):303–5. 15463789 10.1016/0169-4758(93)90131-x

[31] Shokoples SE, Ndao M, Kowalewska-Grochowska K, Yanow SK. Multiplexed real-time PCR assay for discrimination of Plasmodium species with improved sensitivity for mixed infections. J Clin Microbiol. 2009;47(4):975–80. 19244467 10.1128/JCM.01858-08 PMC2668309

[32] Veron V, Simon S, Carme B. Multiplex real-time PCR detection of P. falciparum, P. vivax and P. malariae in human blood samples. Exp Parasitol. 2009;121(4):346–51. 19124021 10.1016/j.exppara.2008.12.012

[33] Zoghi S, Mehrizi AA, Raeisi A, Haghdoost AA, Turki H, Safari R, Survey for asymptomatic malaria cases in low transmission settings of Iran under elimination programme. Malar J. 2012;11:1–10. 22533733 10.1186/1475-2875-11-126 PMC3464154

[34] Atkinson JA, Johnson ML, Wijesinghe R, Bobogare A, Losi L, O’Sullivan M, Operational research to inform a sub-national surveillance intervention for malaria elimination in Solomon Islands. Malar J. 2012;11:1–13. 22462770 10.1186/1475-2875-11-101 PMC3359162

[35] Koukouikila-Koussounda F, Malonga V, Mayengue PI, Ndounga M, Vouvoungui CJ, Ntoumi F. Genetic polymorphism of merozoite surface protein 2 and prevalence of K76T pfcrt mutation in Plasmodium falciparum field isolates from Congolese children with asymptomatic infections. Malar J. 2012;11:1–7. 22463364 10.1186/1475-2875-11-105 PMC3349535

[36] Dal-Bianco MP, Köster KB, Kombila UD, Kun JFJ, Grobusch MP, Ngoma GM, High prevalence of asymptomatic Plasmodium falciparum infection in Gabonese adults. Am J Trop Med Hyg. 2007;77(5):939–42. 17984357

[37] Zhou G, Yewhalaw D, Lo E, Zhong D, Wang X, Degefa T, Analysis of asymptomatic and clinical malaria in urban and suburban settings of southwestern Ethiopia in the context of sustaining malaria control and approaching elimination. Malar J. 2016;15(1):1–9. 27129785 10.1186/s12936-016-1298-2 PMC4851815

[38] Assefa A, Ahmed AA, Deressa W, Wilson GG, Kebede A, Mohammed H, Assessment of subpatent Plasmodium infection in northwestern Ethiopia. Malar J. 2020;19(1):1–10. 32131841 10.1186/s12936-020-03177-w PMC7057598

[39] Tadesse FG, Van Den Hoogen L, Lanke K, Schildkraut J, Tetteh K, Aseffa A, The shape of the iceberg: Quantification of submicroscopic Plasmodium falciparum and Plasmodium vivax parasitaemia and gametocytaemia in five low endemic settings in Ethiopia. Malar J. 2017;16(1):1–11. 28253867 10.1186/s12936-017-1749-4 PMC5335517

[40] Lo E, Yewhalaw D, Zhong D, Zemene E, Degefa T, Tushune K, Molecular epidemiology of Plasmodium vivax and Plasmodium falciparum malaria among duffy-positive and Duffy-negative populations in Ethiopia. Malar J. 2015;14(1):1–10. 25884875 10.1186/s12936-015-0596-4 PMC4340780

[41] Golassa L, Enweji N, Erko B, Aseffa A, Swedberg G. Detection of a substantial number of sub-microscopic Plasmodium falciparum infections by polymerase chain reaction: A potential threat to malaria control and diagnosis in Ethiopia. Malar J. 2013;12(1):1–10. 24090230 10.1186/1475-2875-12-352 PMC3850638

[42] Alemu A, Fuehrer HP, Getnet G, Tessema B, Noedl H. Plasmodium ovale curtisi and Plasmodium ovale wallikeri in North-West Ethiopia. Malar J. 2013;12(1):1–7. 24073668 10.1186/1475-2875-12-346 PMC3849950

[43] Díaz PB, Lozano PM, Rincón JMR, García L, Reyes F, Llanes AB. Quality of malaria diagnosis and molecular confirmation of Plasmodium ovale curtisi in a rural area of the southeastern region of Ethiopia. Malar J. 2015;14(1):1–8. 26383920 10.1186/s12936-015-0893-y PMC4574541

[44] Feleke SM, Brhane BG, Mamo H, Assefa A, Woyessa A, Ogawa GM, Sero-identification of the aetiologies of human malaria exposure (Plasmodium spp.) in the Limu Kossa District of Jimma Zone, South western Ethiopia. Malar J. 2019;18(1):4–9. 31455373 10.1186/s12936-019-2927-3 PMC6712699

[45] Haileselassie W, Zemene E, Lee MC, Zhong D, Zhou G, Taye B, The effect of irrigation on malaria vector bionomics and transmission intensity in western Ethiopia (Parasites & Vectors, (2021), 14, 1, (516), 10.1186/s13071-021-04993-y). Parasites and Vectors. 2021;14(1):1–11. 34620228 PMC8500124

[46] Demissew A, Hawaria D, Kibret S, Animut A, Tsegaye A, Lee MC. Impact of sugarcane irrigation on malaria vector Anopheles mosquito fauna , abundance and seasonality in Arjo - Didessa , Ethiopia. Malar J. 2020;1–8. 32962693 10.1186/s12936-020-03416-0 PMC7510110

[47] Wondwosen B, Birgersson G, Seyoum E, Tekie H, Torto B, Fillinger U, Rice volatiles lure gravid malaria mosquitoes, Anopheles arabiensis. Sci Rep. 2016;6:1–8. 27901056 10.1038/srep37930 PMC5128813

[48] Wondwosen B, Birgersson G, Tekie H, Torto B, Ignell R, Hill SR. Sweet attraction: Sugarcane pollen-associated volatiles attract gravid Anopheles arabiensis. Malar J. 2018;17(1):1–9. 29466989 10.1186/s12936-018-2245-1 PMC5822481

[49] Hawaria D, Getachew H, Zhong G, Demissew A, Habitamu K, Raya B, Ten years malaria trend at Arjo-Didessa sugar development site and its vicinity, Southwest Ethiopia: A retrospective study. Malar J. 2019;18(1):1–11. 31014319 10.1186/s12936-019-2777-z PMC6480840

[50] Kyei-Baafour E, Tornyigah B, Buade B, Bimi L, Oduro AR, Koram KA, Impact of an Irrigation Dam on the Transmission and Diversity of Plasmodium falciparum in a Seasonal Malaria Transmission Area of Northern Ghana. J Trop Med. 2020;2020. 10.1155/2020/1386587 PMC7155757 32308690

[51] Yewhalaw D, Getachew Y, Tushune K, Michael KW, Kassahun W, Duchateau L, The effect of dams and seasons on malaria incidence and anopheles abundance in Ethiopia. BMC Infect Dis. 2013;13(1):1–9. 23566411 10.1186/1471-2334-13-161 PMC3667047

[52] Mawili-mboumba DP, Ndong RN, Rosa NB, Luis J, Largo L, Lembet-mikolo A, Submicroscopic Falciparum Malaria in Febrile Individuals in Urban and Rural Areas of Gabon. Am J Trop Med Hyg. 2017;96(4):815–8. 28219989 10.4269/ajtmh.15-0231 PMC5392626

[53] Almeida ACG, Kuehn A, Castro AJM, Vitor-Silva S, Figueiredo EFG, Brasil LW, High proportions of asymptomatic and submicroscopic Plasmodium vivax infections in a peri-urban area of low transmission in the Brazilian Amazon. Parasites and Vectors. 2018;11(1):1–13. 29558985 10.1186/s13071-018-2787-7 PMC5859403

[54] Ethiopia National Malaria Indicator Survey 2015. Ethiopian Public Health Institute. Addis Ababa, Ethiopia; 2016.

[55] Nigatu W, Woyessa A, Tafesse H, Sisay A, Getachew A, Fentie G, A survey for long-lasting insecticidal net coverage and use in Ethiopia. Ethiop j public Heal nutr. 2019;

[56] Nigatu W, Sisay A, Haile M, Tesfaye G, Awel SA, Wuletaw, A survey on ownership and use of long-lasting insecticidal nets in Ethiopia (2020). Ethiop J Heal Nutr. 2022;5(1):36–43.

[57] Delil RK, Dileba TK, Habtu YA, Gone TF, Leta TJ. Magnitude of malaria and factors among febrile cases in low transmission areas of Hadiya Zone, Ethiopia: A facility based cross sectional study. PLoS One. 2016;1–17. 10.1371/journal.pone.0154277 PMC4854449 27137913

[58] Tilaye T, Tessema B, Alemu K. High asymptomatic malaria among seasonal migrant workers departing to home from malaria endemic areas in northwest Ethiopia. Malar J. 2022;21(1):1–13. 35690823 10.1186/s12936-022-04211-9 PMC9188248

[59] Abossie A, Yohanes T, Nedu A, Tafesse W, Damitie M. Prevalence of malaria and associated risk factors among febrile children under five years: A cross-sectional study in arba minch zuria district, south Ethiopia. Infect Drug Resist. 2020;13:363–72. 32104008 10.2147/IDR.S223873 PMC7012238

[60] Thomas S, Ravishankaran S, Asokan A, Justin NAJA, Kalsingh TMJ, Mathai MT, Socio - demographic and household attributes may not necessarily influence malaria : evidence from a cross sectional study of households in an urban slum setting of Chennai , India. Malar J. 2018;1–11. 29304794 10.1186/s12936-017-2150-z PMC5755004

[61] Mpimbaza A, Achan J. Editorial commentary on : Malaria parasitaemia among long distance truck drivers in the Niger delta of Nigeria. 2012;(February 2021). 10.4314/ahs.v12i2.2 PMC3462537 23056011

[62] Prothero RM. Disease and Mobility : A Neglected Factor in Epidemiology *. 1977;(3). 10.1093/ije/6.3.259 591173

[63] Haile M, Lemma H, Weldu Y. Population movement as a risk factor for malaria infection in high-altitude villages of Tahtay-Maychew District, Tigray, Northern Ethiopia: A case-control study. Am J Trop Med Hyg. 2017;97(3):726–32. 28722582 10.4269/ajtmh.17-0129 PMC5590607

[64] Demissie GD, Ayele TA, Wami SD, Sisay MM, Fetene D, Wolde HF, Low practice of malaria prevention among migrants and seasonal farmworkers in Metema and west Armacheho districts, Northwest Ethiopia. BMC Infect Dis. 2021;21(1):1–9. 33541286 10.1186/s12879-021-05853-x PMC7863355

[65] Lemma W. Impact of high malaria incidence in seasonal migrant and permanent adult male laborers in mechanized agricultural farms in Metema-Humera lowlands on malaria elimination program in Ethiopia. BMC Public Health. 2020;20(1):1–14. 32164610 10.1186/s12889-020-8415-4 PMC7069189

[66] Argaw MD, Woldegiorgis AGY, Workineh HA, Akelom BA, Abebe ME, Abate DT, Access to malaria prevention and control interventions among seasonal migrant workers: A multi-region formative assessment in Ethiopia. PLoS One. 2021;16:1–15. 10.1371/journal.pone.0246251 PMC7901780 33621245

[67] Ayele DG, Zewotir TT, Mwambi HG. Prevalence and risk factors of malaria in Ethiopia. Malar J. 2012;11:1–9. 22691364 10.1186/1475-2875-11-195 PMC3473321

[68] Mohan I, Kodali NK, Chellappan S, Karuppusamy B, Behera SK, Natarajan G, Socio - economic and household determinants of malaria in adults aged 45 and above : analysis of longitudinal ageing survey in India , 2017 – 2018. Malar J. 2021;1–9. 34233690 10.1186/s12936-021-03840-w PMC8265067

[69] Riabinina O, Task D, Marr E, Lin CC, Alford R, O’Brochta DA, Organization of olfactory centres in the malaria mosquito Anopheles gambiae. Nat Commun. 2016;7:1–12. 10.1038/ncomms13010 PMC5063964 27694947

